# Zn_3_N_2_ nanowires: growth, properties and oxidation

**DOI:** 10.1186/1556-276X-8-221

**Published:** 2013-05-10

**Authors:** Matthew Zervos, Chrystalla Karipi, Andreas Othonos

**Affiliations:** 1 Nanostructured Materials and Devices Laboratory, Nanotechnology Research Unit, School of Engineering, University of Cyprus, P.O. Box 20537, Nicosia 1678, Cyprus; 2 Department of Mechanical and Manufacturing Engineering, School of Engineering, University of Cyprus, P.O. Box 20537, Nicosia 1678, Cyprus; 3 Research Center of Ultrafast Science, Department of Physics, School of Physical Sciences, University of Cyprus, PO Box 20537, Nicosia 1678, Cyprus

**Keywords:** Zinc nitride, Nanowires, Optical properties, Electronic structure

## Abstract

Zinc nitride (Zn_3_N_2_) nanowires (NWs) with diameters of 50 to 100 nm and a cubic crystal structure have been grown on 1 nm Au/Al_2_O_3_ via the reaction of Zn with NH_3_ including H_2_ between 500°C and 600°C. These exhibited an optical band gap of ≈ 3.2 eV, estimated from steady state absorption-transmission spectroscopy. We compared this with the case of ZnO NWs and discussed the surface oxidation of Zn_3_N_2_ NWs which is important and is expected to lead to the formation of a Zn_3_N_2_/ZnO core-shell NW, the energy band diagram of which was calculated via the self-consistent solution of the *Poisson-Schrödinger* equations within the effective mass approximation by taking into account a fundamental energy band gap of 1.2 eV. In contrast, only highly oriented Zn_3_N_2_ layers with a cubic crystal structure and an optical band gap of ≈ 2.9 eV were obtained on Au/Si(001) using the same growth conditions.

## Background

III-V compound semiconductor nanowires (NWs) such as InN [1] and GaN [2,3] NWs are currently being investigated in view of their potential application as nanoscale optoelectronic devices for solid state lighting and solar energy conversion. However, their distinct disadvantage is their high cost. Low cost, viable alternatives are therefore desirable and interesting from a technological and fundamental point of view.

To date, there are very few investigations on II-V or IV-V nitrides such as Zn_3_N_2_ and Sn_3_N_4_ NWs, in contrast to the extensive research that has been carried out on their metal-oxide (MO) counterparts, i.e. ZnO [4] and SnO_2_ NWs [5]. More specifically, Sn_3_N_4_ NWs [6,7] with diameters of 100 nm and lengths of 1 to 2 μm were only obtained recently by halide chemical vapour deposition. On the other hand Zn_3_N_2_ NWs have been grown by Zong et al. [8] via the direct reaction of Zn with 250 sccms of NH_3_ at 600°C. The Zn_3_N_2_ NWs had diameters ≈100 nm, lengths between 10 and 20 μm, and were dispersed in Zn. Irregular, Zn_3_N_2_ hollow-like spheres with diameters of ≈3 μm were also obtained under identical growth conditions [9]. Similarly Zn_3_N_2_ nanoneedles have been prepared by Khan et al. [10] and by Khan and Cao [11] who found an indirect energy band gap of 2.81 eV. In contrast, Zn_3_N_2_ layers [12] have been studied in more detail, while p-type ZnO layers have been prepared by thermal oxidation of Zn_3_N_2_[13] which is important since ZnO is usually n-type due to oxygen defects. It should be noted, however, that p-type ZnO layers have also been obtained by nitrogen doping of ZnO using small flows of NH_3_[14,15], which is a topic of active interest since nitrogen is considered to be a shallow-like, p-type impurity in ZnO. In this case, no changes occur in the crystal structure of ZnO. Recently, we carried out a systematic investigation of the post-growth nitridation of ZnO NWs and the changes that occurred in the crystal structure using moderate flows of NH_3_ and temperatures ≤600°C. These favour the formation of ZnO/Zn_3_N_2_ core-shell NWs since we were able to observe not only the suppression of the XRD peaks related to ZnO but also the emergence of new ones corresponding to the cubic crystal structure of Zn_3_N_2_[16]. Higher temperatures, flows of NH_3_ or the inclusion of H_2_ did not lead to the complete conversion of ZnO into Zn_3_N_2_ NWs, instead led to the complete elimination of the ZnO NWs.

Here, we have undertaken an investigation of the synthesis of Zn_3_N_2_ NWs on Si(001) and Al_2_O_3_ via the direct reaction of Zn with NH_3_, thereby complementing our previous study on the post-growth nitridation and conversion of ZnO into Zn_3_N_2_ NWs.

Zn_3_N_2_ NWs with diameters between 50 and 100 nm, lengths of many tens of micrometres, and a cubic crystal structure have been grown on ≈1 nm Au/Al_2_O_3_ between 500°C and 600°C. These exhibited an optical energy band gap of *E*_G_ = 3.2 eV, estimated from steady state absorption-transmission measurements. In contrast, only Zn_3_N_2_ layers were obtained on 1 nm Au/Si(001) using similar growth conditions, which showed photoluminescence (PL) at 2.9 and 2.0 eV with relative strengths depending on their distance from Zn. We compared this with the case of ZnO NWs and discussed the sensitivity of Zn_3_N_2_ to ambient conditions, which is expected to lead to the formation of Zn_3_N_2_/ZnO core-shell NWs, the energy band diagram of which has been determined via the self-consistent solution of the Poisson-Schrödinger equations within the effective mass approximation by taking into account a fundamental energy band gap of 1.2 eV [17].

## Methods

Zn_3_N_2_ was grown using an atmospheric pressure chemical vapour deposition reactor consisting of four mass flow controllers and a horizontal quartz tube (QT) furnace capable of reaching 1,100°C. For the growth of Zn_3_N_2_, Zn pellets (2 to 14 Mesh, 99.9%; Aldrich Company, Wyoming, IL, USA) were cut into small fragments that were weighed individually with an accuracy of ±1 mg. Square pieces of p^+^Si(001) ≈7 mm × 7 mm were cleaned sequentially in trichloroethylene, methanol, acetone, and isopropanol; rinsed with de-ionised water; dried with N_2_ and coated with Au, ≈0.5 to 20 nm by sputtering using Ar at 1 × 10^−2^ mBar after removing the native SiO_2_ in HF. Square samples of Al_2_O_3_ were coated with a thin layer of 0.5 to 1.0 nm of Au after cleaning with the same organic solvents.

After carefully loading 0.2 to 1.0 g of Zn fragments and Au/p^+^Si(001) or Au/Al_2_O_3_ substrates into a boat and recording their positions and relative distances, the boat was inserted into the QT, which was subsequently purged with 450 sccm Ar and 50 sccms H_2_ for 10 min. Then, the temperature was ramped to the desired growth temperature (*T*_G_) using a ramp rate of 10°C/min and flows of 250 to 450 sccms NH_3_, see Table 1. A smaller flow of 50 sccms H_2_ was added in order to eliminate the background O_2_.

**Table 1:** **Temperatures and gas flows used for the growth of Zn**_**3**_**N**_**2 **_**on 1.8 nm Au/Si(001)**

	***T***_**G **_**(°C)**	**NH**_**3 **_**(sccm)**	**H**_**2 **_**(sccm)**
CVD1066	700	250	-
CVD1065	600	250	-
CVD1070	500	450	50
CVD1069	500	450	-
CVD1072	500	250	-
CVD1068	500	50	-

The temperature ramp was 10°C/min, and 0.9 g of Zn was used in all cases. Upon reaching *T*_G_ = 500°C to 700°C, the same flow of NH_3_ and H_2_ was maintained for a further 60 min; after which, the reactor was allowed to cool down slowly for at least 30 min without changing the gas flows.

The sample was always removed when the temperature was lower than 100°C, and the weight of the remaining Zn was measured to find the amount transferred into the gas stream. The QT was changed regularly in order to maintain a clean, high temperature zone for the growth of the Zn_3_N_2_ NWs.

The morphology of the Zn_3_N_2_ NWs was examined with a scanning electron microscope (SEM; TESCAN, Brno, Czech Republic), while their crystal structure and phase purity were determined using a XRD-6000 X-ray diffractometer (Shimadzu Corporation, Tokyo, Japan) with Cu-K*α* source, by performing a scan of *θ* to 2*θ* in the range between 10° to 80°. Finally, PL was measured at 300 K using excitation at *λ* = 267 nm, and the absorption-transmission spectra were taken with a Lambda 950 UV-vis spectrophotometer (Perkin-Elmer Inc., MA, USA).

## Results and discussion

**Figure 1:**
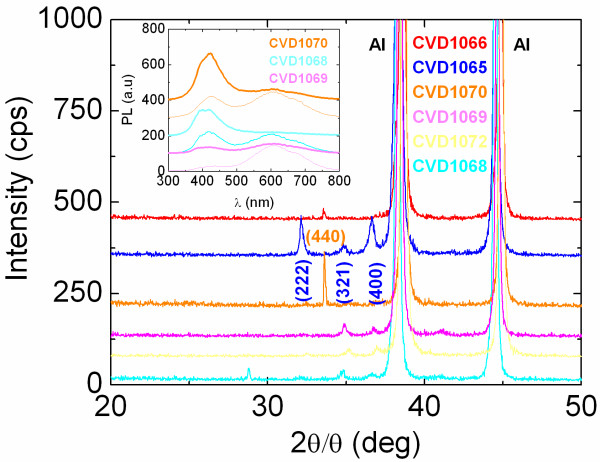
**XRD spectra of the Zn**_**3**_**N**_**2 **_**layers obtained on Si(001) as described in Table **1**.** The peaks belonging to the Al holder have also been identified. The inset shows the room-temperature PL of Zn_3_N_2_ layers grown on 1.8 nm Au/Si(001) at 500°C using 50 sccms NH_3_ (CVD1068 lowest two traces), 450 sccms NH_3_ (CVD1069 mid two traces) and 450 sccms NH_3_:50 sccms H_2_ (CVD1070 top two traces). The bold traces shown in the inset correspond to Zn_3_N_2_ obtained closest to Zn, and the thin ones to Zn_3_N_2_ obtained further donwstream.

We will begin by describing the growth of Zn_3_N_2_ on Au/p^+^Si(001) under different growth conditions listed in Table 1. The reaction of Zn with NH_3_ over Au/p^+^Si(001) between 500°C and 700°C gave very uniform layers with a characteristic yellow or light blue colour. These layers exhibited clear peaks in the XRD, as shown in Figure 1, corresponding to the cubic crystal structure of Zn_3_N_2_. For *T*_G_ = 500°C, we find that small to large flows of 50 to 450 sccms of NH_3_, see Table 1 (CVD1068, CVD1072 and CVD1069), give a set of peaks that are very similar to those of the Zn_3_N_2_ layers prepared by Futsuhara et al. [12], Zn_3_N_2_ NWs of Zong et al. [8,9] and the Zn_3_N_2_ powders of Partin et al. [18]. However, the addition of 50 sccms of H_2_ at the same temperature (CVD1070) led to the complete suppression of all these peaks and the emergence of a single, strong peak at *θ* = 33.3° corresponding to the (440) direction of Zn_3_N_2_. Similar (440) oriented Zn_3_N_2_ layers were obtained at higher temperatures, e.g. 700°C, using moderate flows of 250 sccms of NH_3_ (CVD1066).

All of the Zn_3_N_2_ layers described above exhibited PL emission at 2.9 and 2.0 eV as shown in Figure 1. In particular, the Zn_3_N_2_ layers obtained on Au/Si(001) closest to the source of Zn had the strongest PL at 2.9 eV, while those further downstream from the source of Zn exhibited stronger emission at 2.0 eV. This was observed systematically and is similar to the case of ZnSe, where it was shown that the PL is strong at 450 nm under Zn-rich growth conditions and shifts to 650 nm when the Zn content is lower [19]. It should be noted that the PL at 2.9 eV is comparable to the value of 3.2 eV measured by Kuriyama et al. [20] who prepared Zn_3_N_2_ using NH_3_, while the PL at 2.0 eV is closer to 2.3 eV found by Futsuhara et al. [12]. Different PL and optical energy band gaps have, therefore, been obtained for Zn_3_N_2_ using different growth methods and conditions. Interestingly, the PL peak of the Zn_3_N_2_ layers at 2.9 eV shown in Figure 1 was enhanced by increasing the flow of NH_3_ or by adding H_2_ which also led to a suppression of the side emission at 2.0 eV. The same has also been observed in the growth of GaN NWs or the conversion of *β*-Ga_2_O_3_ into GaN NWs, where the band edge emission at 3.4 eV was boosted using a high flow of H_2_ along with NH_3_ since it passivates surface states or defects within the GaN NWs. Therefore, at first sight, it appears that the main band edge of the Zn_3_N_2_ layers grown here is ≈2.9 eV which is close to the PL of Zn_3_N_2_ layers obtained by a variety of other methods [21]. However, the energy band gap of Zn_3_N_2_ is still a controversial issue, and the optical band gap may not correspond to the fundamental energy gap as will be discussed later in more detail.

**Figure 2:**
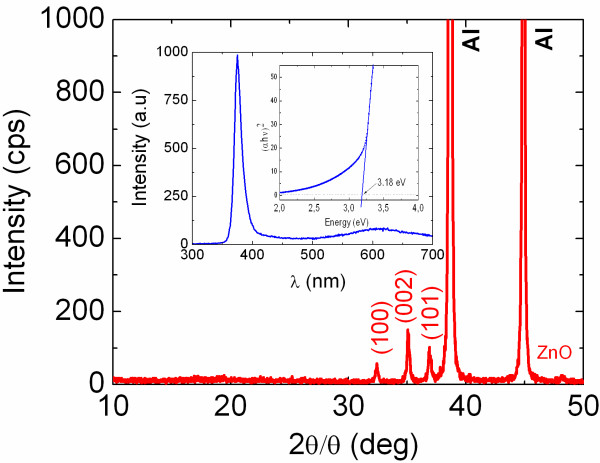
**XRD spectra of ZnO NWs’ lower trace.** Inset shows the PL of the ZnO NWs and square of the absorption versus energy.

No Zn_3_N_2_ NWs were obtained on Au/Si(001) by changing the temperature between 500°C and 700°C, flow of NH_3_, or the thickness of Au between 0.9 and 19 nm while no deposition took place on plain Si(001). This is in direct contrast to the case of ZnO NWs which were obtained readily on Au/Si(001) at 500°C to 600°C by the reaction of Zn with residual O_2_ under an inert flow of 100 sccms Ar by reactive vapour transport or directly on Si(001) without any Au via a self-catalysed vapour solid mechanism. The ZnO NWs showed clear peaks in the XRD as shown in Figure 2, corresponding to the hexagonal wurtzite crystal structure of ZnO.

A typical PL spectrum of the ZnO NWs obtained on Au/Si(001) is shown in Figure 2 with a peak at 390 nm corresponding to 3.2 eV, which is in excellent agreement with the abrupt onset in the absorption measured from ZnO NWs grown on 1.0 nm Au/quartz, shown as an inset in Figure 2. Here, it should be noted that the broad PL of the ZnO NWs at ≈2.0 eV (≡600 nm) is attributed to the radiative recombination of the carriers' occupying defect states that are located energetically in the upper half of the energy band gap, as we have shown in the past for MO NWs such as SnO_2_ and *β*-Ga_2_O_3_ using ultrafast transient absorption-transmission pump-probe spectroscopy [5,22]. This broad PL is not desirable in optoelectronic devices as it represents a competing radiative recombination path which acts to reduce the main band-edge emission.

**Figure 3:**
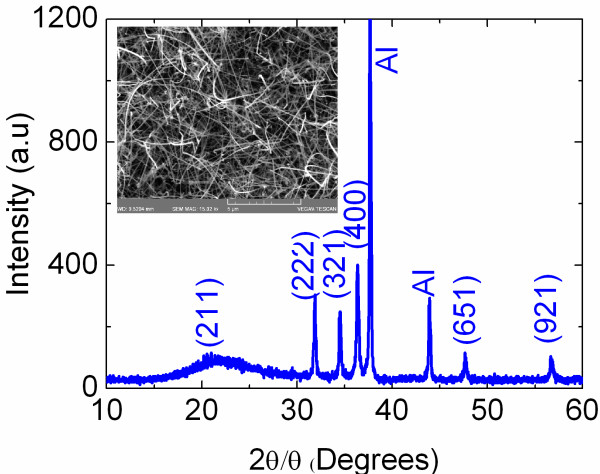
**XRD spectra of the Zn**_**3**_**N**_**2 **_**NWs grown on 1 nm Au/Al**_**2**_**O**_**3 **_**at 600°C under NH**_**3**_**:H**_**2**_**.** Inset shows the SEM image of Zn_3_N_2_ NWs.

While we did not obtain any Zn_3_N_2_ NWs on Au/Si(001), we found that the reaction of Zn with 250 to 450 sccm NH_3_ including 50 sccm H_2_ over 1.0 nm Au/Al_2_O_3_ at temperatures between 500°C and 600°C led to the growth of Zn_3_N_2_ NWs with diameters of 50 to 100 nm and lengths of many tens of micrometres as shown in Figure 3. We should note that the Zn_3_N_2_ NWs probably follow a vapour-liquid-solid-like mechanism similar to the case of GaN NWs, since no deposition occurred on plain Si(001) or Al_2_O_3_. The XRD of the Zn_3_N_2_ NWs, also shown in Figure 3, is similar to that of the Zn_3_N_2_ layers prepared on Au/Si(001). In addition, we observed new peaks which are characteristic of Zn_3_N_2_ and, more importantly, do not belong to ZnO.

**Figure 4:**
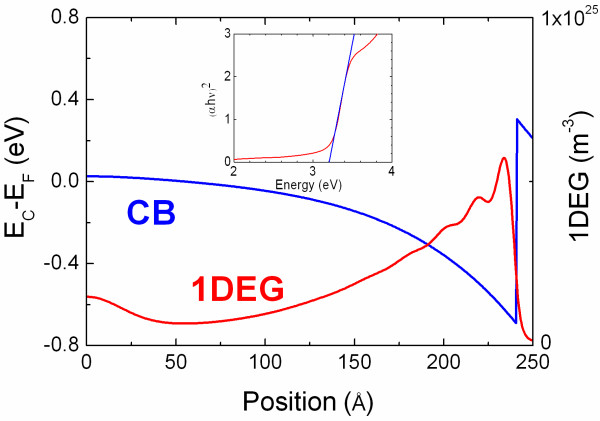
**Self-consistent conduction-band edge potential with respect to the Fermi level.***E*_C_ − *E*_F_ (*E*_F_ = 0 eV) versus radial position for a 50-nm diameter Zn_3_N_2_/ZnO core-shell NW. The core has a radius of 24 nm. Inset shows the absorption squared versus energy for the Zn_3_N_2_ NWs grown on 1 nm Au/Al_2_O_3_.

The absorption-transmission spectrum of the Zn_3_N_2_ NWs that were grown on 1.0 nm Au/Al_2_O_3_ was measured with a Perkin-Elmer Lamba 950 used to determine the optical band gap *E*_OP_ according to α*hν* ∝ (*hν* − *E*_OP_)^*n*^ by extrapolating the linear portion of the curve to zero absorption, where *hν* is the photon energy and *n* = 1 / 2 for direct transitions. A plot of the square of absorption versus energy for the Zn_3_N_2_ NWs grown here is shown as an inset in Figure 4 from which we find that *E*_OP_ = 3.2 eV which is consistent with the PL of the Zn_3_N_2_ NWs of Zong et al. [8] and the PL emission of the Zn_3_N_2_ layers shown in Figure 1, as well as with the predictions of Long et al. [23] who suggested that optical gap energies measured in the range 2.12 to 3.2 eV correspond to band-to-band transitions.

However, according to the *ab initio* electronic structure calculations of Li et al. [17], the fundamental gap of Zn_3_N_2_ is 1.17 eV which is in agreement with the results of Suda and Kakishita [24] who found that the energy gap of polycrystalline Zn_3_N_2_ layers grown by molecular beam epitaxy on quartz is ≈1.0 eV and explained that large blue shifts of the *E*_OP_ are due to the *Burstein-Moss* shift. In addition, the large carrier densities of 10^19^ to 10^20^ cm^−3^ measured by Suda and Kakishita [24] were attributed to oxygen contamination.

We ought to mention here that the growth conditions for the Zn_3_N_2_ NWs gave Zn_3_N_2_ layers on Au/Si(001), not ZnO NWs which would have been obtained if the oxygen background was substantial. Since no ZnO NWs were obtained, the oxygen background under the conditions used for the growth of the Zn_3_N_2_ is negligible, especially under the presence of H_2_.

In short, it is unlikely that the Zn_3_N_2_ NWs contain O from the main gas stream, while it is also unlikely that they contain O from the Al_2_O_3_ bonds which are extremely stable at 500°C to 600°C. However, it is known that Zn_3_N_2_ is sensitive to moisture in air [25], so one expects the formation of a Zn_3_N_2_/ZnO core-shell NW, but not the complete conversion of Zn_3_N_2_ into ZnO NWs since we observed peaks that are clearly related to Zn_3_N_2_ after exposure to air, as shown in Figure 3. Despite intensive investigations on the properties of ZnO, little is known about its surface properties. While a few claim that the Fermi level is pinned above the conduction band edge [26], others claim that the Fermi level is pinned below the conduction band edge [27]. Here, we take the Fermi level to be located below the conduction band edge as in the case of n*-*type ZnO NWs [28]. This is also in accordance with Long et al. [23] who suggested that Zn_3_N_2_ with N substituted by O (O_N_) is more stable than Zn replaced by O (O_Zn_) or interstitial O (O_I_). In the case of O_N_, the Fermi level locates near the bottom of the conduction band, but in the cases of both O_Zn_ and O_I_, the Fermi level is pinned around the top of the valence band [23]. In other words, interstitial oxygen gives p-type Zn_3_N_2_, but since it is not energetically favourable, we expect to have the formation of n-type ZnO shell at the surface which surrounds an n-type Zn_3_N_2_ core.

The energy band diagram of a 50-nm diameter Zn_3_N_2_/ZnO core-shell NW determined from the self-consistent solution of the Poisson-Schrödinger equations (SCPS) in cylindrical coordinates and in the effective mass approximation is shown in Figure 4. In such a calculation, Schrödinger's equation is initially solved for a trial potential *V*, and the charge distribution *ρ* is subsequently determined by multiplying the normalised probability density, ∣*ψ*_*k*_∣^2^, by the thermal occupancy of each sub-band with energy *E*_k_ using Fermi-Dirac statistics and summing over all *k*. The Poisson equation is then solved for this charge distribution in order to find a new potential V′, and the process is repeated until convergence is reached. A detailed description of the SCPS solver is given elsewhere [29,30].

In this calculation, we have taken into account the effective mass *m*_e_^*^ = 0.29 m_o_ and static dielectric constant *ϵ*_r_ = 5.29 of Zn_3_N_2_[24,31], as well as *m*_e_^*^ = 0.24 m_o_ and *ϵ*_r_ = 8.5 for ZnO [32,33]. In addition, we have taken into account the energy band gap of Zn_3_N_2_ to be 1.2 eV [17,24] and the Fermi level to be pinned at 0.2 eV below the conduction band edge at the ZnO surface [28]. A flat-band condition is reached at the centre of the Zn_3_N_2_/ZnO NW, and a quasi-triangular potential well forms in the immediate vicinity of the surface, holding a total of eight sub-bands that fall below the Fermi level. The one-dimensional electron gas (1DEG) charge distribution is confined to the near-surface region, has a peak density of 5 × 10^18^ cm^−3^ (≡5 × 10^24^ cm^−3^), as shown in Figure 4, and a 1DEG line density of 5 × 10^9^ m^−1^.

Optical transitions in this case will occur between the valence band and conduction band states residing above the Fermi level similar to the case of InN [1]. Consequently, the optical and transport properties will depend strongly on the surface properties, but further investigations are required in order to follow up and confirm that the energy band gap of Zn_3_N_2_ is 1.2 eV [17,24] and if it is possible to obtain a p-type ZnO by thermal oxidation of the n-type Zn_3_N_2_ NWs which would be important for device applications.

## Conclusion

Zn_3_N_2_ NWs with diameters of 50 to 100 nm and a cubic crystal structure have been grown on 1 nm Au/Al_2_O_3_ between 500°C and 600°C under a steady gas flow of NH_3_ containing H_2_. These exhibited a large optical band gap of 3.2 eV determined from absorption-transmission steady state spectroscopy. The surface oxidation of Zn_3_N_2_ is expected to lead to the formation of a Zn_3_N_2_/ZnO core-shell NW, the energy band diagram of which was calculated via the self-consistent solution of the Poisson-Schrödinger equations within the effective mass approximation by taking into account a fundamental energy band gap of 1.2 eV for Zn_3_N_2_. Uniform Zn_3_N_2_ layers were obtained on Au/Si(001), while no deposition took place on plain Si(001), in contrast to the case of ZnO NWs which grow with or without a catalyst on Si(001) via the reaction of Zn with O_2_.

## Competing interest

The authors declare that they have no competing interests.

## Authors’ contributions

MZ and CK carried out the synthesis, scanning electron microscopy and X-ray diffraction. The optical properties were measured by AO. The calculations were carried out by MZ who was also wrote the manuscript. All authors read and approved the final manuscript.
